# Establishment of a perinatal mental health programme in a portuguese public hospital

**DOI:** 10.1192/j.eurpsy.2022.2236

**Published:** 2022-09-01

**Authors:** T. Reis, D. Leite, M. Serra

**Affiliations:** 1 Hospital Espirito Santo de Évora, Departamento De Psiquiatria E Saúde Mental, ÉVORA, Portugal; 2 Hospital Espirito Santo de Évora, Departamento De Psiquiatria E Saúde Mental, Évora, Portugal

**Keywords:** programme, women, perinatal mental health, intervention

## Abstract

**Introduction:**

The perinatal period constitutes a unique individual and family experience, involved in multifaceted transformations and adaptations at the physical, psychological, social, and emotional levels. This is the period in women’s life cycle where there is a higher risk for the development of mental illness.

**Objectives:**

To introduce the perinatal mental health programme of the Hospital do Espírito Santo de Évora. The main objective is to structure an intervention with the woman and her support network to promote healthy parenting.

**Methods:**

Implementing secondary and tertiary intervention approaches in a general and public hospital in the Alentejo region of Portugal. The programme is composed of the following components and domains of intervention in the pre-conception, pregnancy, and post-partum periods: individual consultation; brief intervention consultation; mindfulness sessions in the immediate postpartum period; home-based interventions; empowerment interventions for hospital and community healthcare professionals.

**Results:**

It is expected that the project will result in a multidisciplinary approach to perinatal mental health, with significant impact, improved perinatal mental health of the women integrated in the project, as well as improved level of satisfaction in the provision of care in the woman/family.

**Conclusions:**

Considering the prevalence and impact of mental health issues in the perinatal period, it is desirable to structure interventions with a holistic and multidisciplinary approach. Perinatal mental health should be prioritized during the entire process of pregnancy and postnatal period. A network of primary and secondary care systems may allow mitigating and/or overcoming vulnerabilities.

**Disclosure:**

No significant relationships.

